# Older Adults’ Attitudes Toward Ambulatory Technology to Support Monitoring and Coaching of Healthy Behaviors: Qualitative Study

**DOI:** 10.2196/10476

**Published:** 2019-03-12

**Authors:** Miriam Cabrita, Monique Tabak, Miriam MR Vollenbroek-Hutten

**Affiliations:** 1 eHealth Group Roessingh Research and Development Enschede Netherlands; 2 Biomedical Signals and Systems Group Faculty of Electrical Engineering, Mathematics and Computer Science University of Twente Enschede Netherlands

**Keywords:** wearable technology, telemedicine, independent living, healthy aging, nutritional status, cognitive function, physical activity

## Abstract

**Background:**

Prevention of functional decline demands a holistic perspective of health management. Older adults are becoming avid users of technology; however, technology is not yet largely used in supporting self-management of health in daily life. Previous research suggests that the low adherence to these technologies is likely to be associated with the fact that opinions and wishes of the older population are not always taken into consideration when designing new technology.

**Objective:**

The aim of this study was to investigate the attitudes of older adults living independently regarding technology to support healthy behaviors, addressing nutrition, physical and cognitive function, and well-being.

**Methods:**

In-depth semistructured interviews were performed with 12 older adults addressing 4 themes: (1) current practices in health management, (2) attitudes toward using technology to support health management, (3) wishes from technology, and (4) change in attitudes after actual use of technology. The fourth theme was investigated with a follow-up interview after participants had used a step counter, a smart scale, and a mobile app for 1 month. Data collected were analyzed using inductive thematic analysis.

**Results:**

Participants were active in self-managing their health and foresaw an added value on using technology to support them in adopting healthier behaviors in everyday life. Attitudes and wishes differed considerably per health domain, with cognitive function being the most sensitive topic. Fears from technology mentioned were attention theft, replacement of human touch, and disuse of existing abilities. Poststudy interviews suggest that attitudes toward technology improve after a short period of use.

**Conclusions:**

Technology to support aging in place must target health literacy, allow personalization in the design but also in the use of the technology, and tackle existing fears concerning technology. Further research should investigate the effect of these strategies on the adherence to technology to be used in daily life. We outline a set of recommendations of interest to those involved in developing and implementing technology to support aging in place, focusing on acceptance, barriers, and ethical concerns.

## Introduction

The increase of life expectancy is one of the factors contributing to the growing proportion of the population aged above 60 years in developed countries. However, these extra years are not always perceived as *healthy years* with the World Health Organization stressing the need to *add health to years* [1]. One possible solution is by empowering older adults to self-manage their health and consequently prevent functional decline. Technology can play a crucial role here. In fact, in the last decade, we have experienced a growing interest in the research and development of technology for the use of older people. Ambulatory technology, that is, technology that is used to assess or intervene during daily life experiences, in particular, can provide continuous real-time information on the health status of the older individual, detect changes over time, and promote healthy behaviors to prevent or early detect functional decline.

Functional decline can result from a sudden event (eg, a fall resulting in hip fracture) or from a complex interaction between multiple factors combining, among others, lifestyle and presence of chronic diseases. Functional decline can also be a slow process that develops in daily life. In other words, prevention of functional decline requires a holistic approach to health, rather than focusing on one specific health domain. Hence, technology to prevent, detect, or even reverse functional decline should take a multidimensional perspective of health and should be integrated into the daily life of the users, in this case, older adults.

The adoption rates of such information technologies by older adults are growing [2], and contrary to popular belief, this age group is in general open-minded toward electronic health [3]. However, there are still well-known barriers constraining the adoption and acceptance of technology. Among the critical barriers to the adoption of technologies by older adults are privacy concerns, ease of usability for daily use, and the belief that the technology is not necessary, that is, the perception of no need [4].

One way to prevent these barriers is to include older adults in all phases of development of the new product or service, that is, participatory design. In fact, a review from Piau et al reveals that inadequate comprehension of user needs is a major issue compromising acceptance of technology [5]. Prioritization of needs and wishes of older adults to improve adherence to and acceptance of technology is mentioned not only by the older adults themselves [6] but also by several stakeholders involved in the development and deployment of technologies, such as care professionals, technologists, and policy makers [7,8]. However, despite the current knowledge on the importance of involving older adults in all phases of research and development, studies investigating the wishes of older adults with regard to technology to prevent functional decline are scarce. Furthermore, most user-centered design studies are performed envisioning the development of a product targeting 1 or 2 health domains [9,10], instead of the holistic approach required by the definition of functioning. In this work, we investigate, through semistructured interviews, the current practices in self-management and the attitudes as well as the wishes of older adults concerning technology to be used in daily life supporting their health management and preventing functional decline. Given the multidimensional definition of functioning, we take a holistic perspective of health, considering each one of the following domains in particular: nutrition, cognitive function, physical function, and well-being.

The literature has shown that the expected effort from using technology can decrease after a short period of use [11,12]. In addition, older adults perceive that sometimes they need a small nudge to use technology [13]. Consequently, we deployed a case study in which participants are provided with an example of ambulatory technology, and we investigated whether their attitudes toward monitoring health with technology change after the actual use. Thus, our study addresses 4 main points:

1. Current practices in health management
2. Attitudes toward using technology in health management
3. Wishes and expectations from technology
4. Attitudes toward using technology in health management after actual use.

This study extends the existent work on understanding the barriers and motivators to use technology among community-dwelling older adults [4,13] by looking at technology to support empowerment of older adults in managing their health, from the perspective of older adults. With this study, we aim to provide insights to researchers, clinicians, and all those interested in developing technology contributing to the improvement of acceptance and adherence to technology-based interventions to be implemented in the daily life of older adults.

## Methods

### Participants

A total of 23 older adults were recruited in local information markets to promote healthy behaviors in the region of Overijssel, the Netherlands, as well as in information sessions given to participants in the European Project FP7-Personalised ICT Supported Service for Independent Living and Healthy Ageing (PERSSILAA) [14] between December 2015 and January 2016. Within the PERSSILAA project, a multidisciplinary and international consortium developed, implemented, and evaluated a novel service model to screen for and prevent frailty among community-dwelling older adults [15]. This new service model was implemented in Italy (Campania) and the Netherlands (Overijssel), counting for involvement of more than 7000 older adults between 2013 and 2016.

All those interested in participating in this study received a letter explaining the research in more detail via post or email. Overall, 12 older adults confirmed interest in participating in the research and were invited for an interview at Roessingh Research and Development. The research was explained by the interviewers; the potential participants were given time to ask questions and afterward provided written informed consent. The ethical review board of the University of Twente approved the study. This study did not require approval of the medical ethical review board, according to European regulations, as all respondents were competent individuals and this study did not involve any interventions or treatments.

### Semistructured Interview

An individual interview scheme was chosen as this method provides freedom and openness to explore the opinion of the participants. Each individual interview started with an introductory session in which 2 interviewers informed the participant that the interview would be audio recorded and that it would take approximately 1 hour. One of the interviewees was mostly engaged in the conversation and the other was responsible for taking notes and to intervene whenever necessary. The interview was divided into 5 main topics: general health management, nutrition, cognition, physical function, and well-being.

The general health management questions served the purpose to create a context to go further with the other topics by opening the interview. Current practices in health management, attitudes toward monitoring health with technology, and wishes from technology were assessed with the following open questions for each one of the health domains addressed: (1) *What are you currently doing to manage your physical function, cognitive function, nutrition, and well-being?* (2) *What is your attitude toward monitoring physical function, cognitive function, nutrition, and well-being?* and (3) *What are your wishes and expectations from technology to monitor physical function, cognitive function, nutrition, and well-being?* The well-being scale did not have a question regarding current self-management practices.

### General Health Assessment

A general health assessment was performed to obtain an overview of the health status of the participants. Participants were given the choice to answer the questionnaire right after the end of the interview or to take it home and answer at a time of their convenience and send it to the research facilities via standard post. General frailty was assessed with the Groningen Frailty Indicator [16], a 15-point yes-no questionnaire exploring physical, cognitive, social, and psychological components of frailty, with a score equal to or above 4 being regarded as moderate to severely frail (referred to as *decline* in Table 1). Physical limitations were assessed using the physical functioning scale of the Short Form-36 Health Survey [17], a questionnaire with a range between 0 (limited) and 100 (not limited) and with a value higher than 61 being an indication of physical decline. Cognitive function was assessed with the AD8 Dementia Screening scale [18], with a score higher than 2 being an indicator of cognitive decline. Finally, the nutritional status was assessed with the Mini Nutritional Assessment Scale [19], with a total score in the questionnaire between 7 and 23.5 being an indicator of malnutrition (referred to as *decline* in Table 1). This frailty screening method was used with more than 10,000 people in Italy and the Netherlands during and after the European project PERSSILAA [15].

### Actual Use of Technology

At the end of the interview, participants were lent a mobile phone, a Fitbit Zip step counter, and a smart scale Withings 30. The purpose was to let the older adults experience simple technology to monitor parameters from 3 health domains using technology.

Regarding the physical activity domain, the participants received instructions to wear the step counter in the pocket to assess the number of steps throughout the day. Feedback on physical activity was provided on the step counter and also on the screen of the mobile phone, using the Activity Coach app. This app has been used in interventions to promote physical activity among several clinical populations, such as cancer survivors [20,21] and patients suffering from chronic pulmonary obstructive disease [22]. In the mobile phone, participants received feedback on the number of steps they took in the current day and the distribution of steps in the current day per hour and during the last week per day. Participants could also see a representation of how far they were from reaching the daily goal. The daily step goal was set to 7500 steps, following recent research [23]. Participants were told that this goal could be changed upon request.

On the nutrition domain, a smart scale was used to monitor weight. The measurements on the smart scale were made available in real time on the Activity Coach app, with an indication of variation of weight and body mass index since the last measurement and also how (un)healthy the values measured were.

Well-being was assessed in de mobile app. Participants were asked at the end of every day (at 8.30 pm) to which extent they experienced 6 discrete positive emotions (*joy, amusement, awe, love or friendliness, interest, and serenity*) and to rate it on a Likert scale ranging from 1 (*not at all*) to 7 (*very intense*). The selection of positive emotions was based on the modified Differential Emotions Scale [24] and covered the full arousal or activation dimension.

The technology was carefully explained to the participants. The researchers encouraged the participants to contact the research team in case of any question or doubt. Participants were asked to use the technology at their own pace during 4 weeks. At the end of this period, a new interview was performed to assess the experience of the participants and evaluate whether their attitude toward using technology to monitor their health in daily life had changed.

### Study Setting

**Figure 1:**
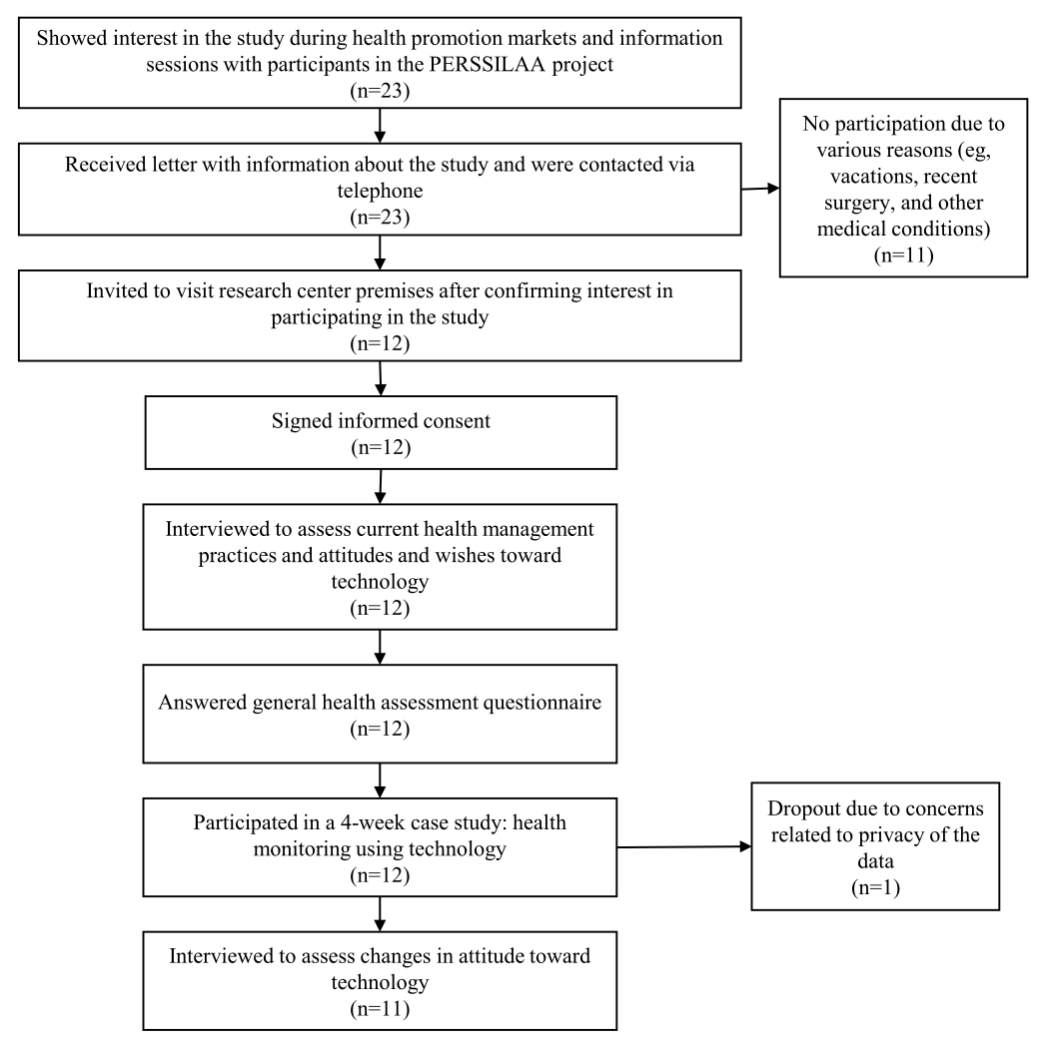
Flow diagram with all phases of the study and number of participants at each phase. PERSSILAA: Personalised ICT Supported Service for Independent Living and Healthy Ageing.

This study took place between March and June 2016. The flow diagram in Figure 1 illustrates the several steps of the study and the total number of participants at each stage. Of the 23 older adults approached, 12 showed interest in participating in the study when contacted to schedule the interview. Reasons to not participate were being abroad for the duration of the study or health-related (eg, recent surgery). Both interviews were conducted face-to-face, at the premises of the research center, and with 2 researchers and 1 participant at a time. One participant dropped out of the study during the case study because of concerns regarding the privacy of the data collected. The researchers assured the participant that the data collected were treated following international guidelines, but nevertheless, the participant felt overwhelmed with the amount of data being collected on a daily basis. At the end, the concerns were not related to the study per se. This participant did not answer the questionnaire regarding current health status but gave permission to analyze the data collected until the moment of dropout in the analysis of the results.

### Data Analysis

The interviews were audio recorded and transcribed verbatim. The transcripts were first categorized using a concept-driven approach by 2 researchers, considering the categories: *current practice, attitudes toward health management,* and *wishes from technology*. A more detailed categorization in subthemes was performed using inductive thematic analysis [25]. An iterative process was taken until eliciting the final codes. The data analysis was supported by the use of the qualitative data analysis software Atlas.ti 7.0 (Scientific Software Development GmbH).

## Results

### Participants

A total of 12 community-dwelling older adults (aged 65-78 years) participated in the interviews and 11 concluded the case study. Table 1 provides a summary of the demographic characteristics and health status of the participants at the moment of the interview. Overall, 7 out of the 12 participants were women and 8 lived with someone else (in most cases with the partner or spouse). Most of the participants were robust on the frailty scale, and the highest percentage of limitations was found on the physical functioning scale (only 2 out of 11 participants had limitations). Regarding previous experience with ambulatory technology, 8 of the 12 participants had a smartphone and 5 of them considered themselves advanced users, as they used the device for Web browsing and emailing. None of the participants had previous experience with ambulatory monitoring of physical activity or smart scale devices.

### Current Practices in Health Management

Overall, 11 out of 12 interviewees mentioned general health practices from the physical domain (eg, sports), and half of the interviewees mentioned paying attention to eating habits on a daily basis (eg, avoiding candies). One interviewee mentioned sleep hygiene (eg, always sleep for 8 hours per night) and another mentioned mental well-being (eg, by performing pleasurable activities).

When asked about the reasons why it is important to keep track of their health, 9 participants mentioned their current medical situation, often suffering from at least one chronic condition. In addition, 4 interviewees mentioned that they want to keep doing their daily activities independently. Participants showed to be concerned about the fact that if they stop living normal life, they might not come back to current activity (eg, “Because if you start sitting still, you will rust.” [a 66-year-old female]).

**Table 1:** Demographic characteristics of the participants (age, gender, living situation, and education) and other parameters regarding lifestyle (smoking status) and current health status (general frailty, nutrition, cognitive function, and physical function).

Characteristic		Statistics
Age (years), mean (range)		69 (65-78)
**Gender, n (%)**		
	Female	7 (58)
	Male	5 (42)
**Living situation, n (%)**		
	Alone	3 (25)
	With someone else	8 (67)
	Missing	1 (8)
**Education, n (%)**		
	Elementary school	1 (8)
	High school	3 (25)
	Vocational school	6 (50)
	University	1 (8)
	Missing	1 (8)
**Smoking, n (%)**		
	Smoker	2 (17)
	Nonsmoker	9 (75)
	Missing	1 (8)
**General frailty, n (%)**		
	Decline	3 (25)
	Robust	7 (58)
	Missing	2 (17)
**Nutrition, n (%)**		
	Decline	0
	Robust	11 (91)
	Missing	1 (8)
**Cognitive function, n (%)**		
	Decline	1 (8)
	Robust	9 (75)
	Missing	2 (17)
**Physical function, n (%)**		
	Decline	2 (17)
	Robust	9 (75)
	Missing	1 (8)
Body mass index, mean (range)		25.3 (17.4-36.1)

Another factor often mentioned was the fast decline or even sudden death of beloved ones in the surroundings of the interviewees and how that affects the self-perception of health. Finally, 1 interviewee mentioned that, with the amount of information available nowadays, it is imprudent (*silly*) if one does not take care of his own health.

When asked about the motivation to keep track of their health, none of the participants referred *to avoid disease*. Instead, the functional perspective of health was very present as participants said they wanted to be healthy to keep doing their daily activities independently.

In general, interviewees were aware that their health status is changing as they get older and wanted to adopt measures to slow down this process, such as monitoring their current health status and training to improve their general functioning (eg, “Health is your biggest treasure!” [a 68-year-old female]).

#### Nutrition

Overall, 11 out of 12 interviewees stated the adoption of healthy practices in their daily diet, such as cooking with low salt/sugar/fat/carbohydrates, taking small portions, and including vegetables in all warm meals. Only 1 interviewee mentioned not paying attention to the daily eating and added that he or she only cooks warm meals when accompanied, otherwise breakfast, lunch, and dinner would consist of bread. From the analysis of the interviews, it is clear that the eating habits are influenced not only by the medical background of the interviewee but also by the medical background of the spouse, as a couple is likely to cook and eat together. It is also noteworthy that the interviewee who would need to take care of daily diet the most (because of being overweight) was the only one who refers not paying attention to eating habits.

#### Cognitive Function

When asked about the current practices to self-manage cognitive function, most of the interviewees did not understand the concept. After hints from the interviewers, the interviewees mentioned that, in fact, although unaware, they were regularly training their cognition. Examples of activities mentioned were *puzzles* (n=6), *read books and newspapers* (n=6), and *play computer games* (n=2). Interviewees were aware that their memory was declining with age and showed to be concerned about that fact. For those who had relatives or friends who suffer, or have suffered, from conditions as Alzheimer disease, or similar, cognitive decline was perceived as a very sensitive topic to talk about.

#### Physical Function

Interviewees showed to be more aware of healthy behaviors concerning prevention of physical function decline than of any other health domain. A total of 7 out of the 12 interviewees practiced sports at least twice a week. Sports mentioned included tennis, golf, swimming, and fitness. In addition, 5 participants mentioned that continuing to do the household chores by themselves helped them to feel active. Other physical activities mentioned were dancing, volunteer work, and recreational biking or walking. Furthermore, 7 participants mentioned to use the bike for everyday commuting and only preferring the car or bus when the weather is bad. Noteworthy is that one of the interviewees mentioned that most of the daily physical activity comes from his or her role as an informal caregiver, considering that he or she needs to take care of everything for the spouse, whenever needed. In addition, 2 interviewees mentioned to be goal-oriented persons, and therefore, they could not think of biking, walking, or exercising without a meaningful activity. Participants reported that they felt more energetic when they are more active on a daily basis.

The medical background had a strong influence in the 3 health domains investigated. For example, in the physical function domain, for some participants, the medical condition was a motivation to be more active as, for example, diabetic patients knew that an active lifestyle would help in controlling insulin levels. For other participants, the medical background represented a constraint on becoming active, as for patients with chronic obstructive pulmonary disease or with cardiovascular diseases.

Compensation strategies were mentioned related to daily diet (nutrition domain) and physical function. For example, 1 participant said:

[My wife] cooks some special kind of bacon, it’s very nice and spicy, a good feeling; when I eat it, I don’t touch the sweets.

78-year-old male

### Attitudes Toward Using Technology in Health Management

#### Nutrition

All interviewees recognized that it is important to keep track of the daily diet. However, 6 out of 12 participants claimed that they would not use a website or app to monitor their eating habits. Some participants said that they monitor their food intake by themselves and do not need technology to help with it; others believed that it would be too time-consuming to log everything they eat throughout the day in an app or website. One participant said he or she would prefer to talk to someone about the topic rather than to use technology. In addition, 2 participants kept a food diary log for a couple of years because of their diabetic condition.

#### Cognitive Function

All participants recognized the importance of keeping good cognitive functioning for performing daily activities independently. None of the participants stated clearly that they would not like to use an app or website to train their cognitive function. Moreover, 3 interviewees stated that they were afraid to get an overview of their cognitive function over time as they would not want to be confronted with a decline:

Well, I’m actually a bit afraid. [...] I’m doing everything to prevent it, but when I get it, I prefer not to be confronted with it.

69-year-old female

Reasons for this fear are close cases of dementia (eg, Alzheimer disease) or their own medical history.

#### Physical Function

When first asked, most of the interviewees were not open to the idea of monitoring physical activity. After explanation, 5 out of the 12 participants continued saying that they did not find it important to monitor physical activity with technology, as, according to them, they felt when they were active enough or when they were not. Due to that, participants perceive that they do not need to see the physical activity level in any technology:

Look, when I’m fit and active, I feel good. Well, and I can feel that myself, I don’t need to see that on one of the computers, “you did this and that...”

72-year-old female

One participant mentioned that monitoring physical activity with technology would likely make him or her less attentive to the own body. At the end, 6 participants mentioned that they would maybe use an app or website to monitor their physical activity.

#### Well-Being

Participants were asked to think specifically of daily well-being, as in how they feel on a daily basis. Overall, 8 participants found it important to keep track of their well-being on a daily basis; 4 participants did not see any added value in monitoring well-being. One participant mentioned that she does not ever reflect on their own well-being:

No, no, no, but that’s something like, I have never, you notice that, today I feel a bit better than yesterday. I have never thought about that, because the day starts with fixed routines, and you will see how it ends.

72-year-old female

Overall, 1 participant perceived emotional information too personal and would only share it with a specialist. In addition, 6 participants mentioned that they would at least try the app to monitor well-being and 2 interviewees totally rejected the idea of it.

### Wishes and Expectations From Technology

#### Nutrition

In general, interviewees were not aware of the possibilities provided by technology. In this way, most of the suggestions came from the interviewers to which the participants provided vague answers, such as “Maybe that could be interesting.” After suggestions from the interviewers, 3 participants said it could be good to obtain an overview of the daily food intake. The opinions of participants diverged regarding the possibility to monitor caloric and nutritional intake and obtain recommendation of healthy recipes. Reasons for not wanting such services were related to the reluctance to break old routines of eating.

Nevertheless, some participants themselves expressed the wish to have access to a website or app to monitor the nutritional or caloric intake. Moreover, 4 interviewees said they would wish to receive recommendation on healthy recipes tailored to their medical background and needs. In addition, 1 participant said he or she would like to share his or her own knowledge on nutrition with other people. Furthermore, 1 participant said he or she would rather talk to someone or follow a course than use technology.

Participants with a positive attitude toward the use of technology in daily life said that they would likely adapt their behavior to the recommendations or the overview provided by an eventual system.

#### Cognitive Function

The wishes from technology to monitor and train cognitive function were very conflicting. Although 7 interviewees stated that they would like to train cognitive function in a fun way, 3 interviewees clearly stated that they did not like games and would not want to play. In addition, 1 participant mentioned that, when existing, exercises should be short and vary over time to remain engaging. Another interviewee mentioned that the exercises should be tailored to the current cognitive level of the individual. Moreover, 3 participants said that they would like to be able to train their cognitive functioning with technology but would not want to see an overview:

I’m just afraid of it, you know? [...] Well, because it appeared that, with diabetic people, dementia occurs much more often. And then I think, oh boy...and I’m just hiding that. I am telling you now, but yes, that is a fear that I have.

68-year-old female

On the contrary, 3 interviewees mentioned that they would like to receive feedback on their progress over time. In general, participants recognized the importance of preserving cognitive function at old age:

Well, I think you should train it one way or the other, and whether that’s done with some puzzles, with an app or through something else, I don’t care, but it has to happen.

66-year-old male

#### Physical Activity

Participants expressed stronger wishes related to monitoring physical activity with technology than to any other health domain. Interviewees would like to see the distribution of physical activity throughout the day in terms of intensity of activity and number of steps (n=6), the distance walked and biked at the end of the day (n=2), as well as the quantity of calories burnt (n=2). Participants would also like to receive personalized coaching in terms of daily physical activity goals and functional physical exercises tailored to their health, age, and gender (n=3). In addition, 1 interviewee would like to be able to set his or her own activity goals. Another participant mentioned that he or she would likely be motivated by a gamified coaching system in which he or she could receive points every time the goals were reached. One participant would like to have a distinction between activities performed indoors and outdoors. Finally, 1 participant would not like to see the amount of time spent inactive as it would probably be too confronting.

Most participants mentioned that such technology would be very important to make them aware about their actual activity level in comparison with their peers—“am I really more or less active than other people of the same age or medical condition?”—and that they would adapt their behavior to the feedback received. A recurrent worry from 1 participant was the fact that technology could cause attention theft.

#### Well-Being

It was very difficult for interviewees to imagine how it would be to use an app to monitor well-being in daily life. After some hints from the interviewers, 1 participant said he or she would like to compare the well-being of different days. In addition, 4 participants said that they would like to obtain an overview over time to help understand what influences their well-being from day to day. One participant would like to see a figure comparing physical activity and well-being, and another participant would like to receive advice on how to improve well-being. One participant said that to talk about well-being, he or she had to feel sympathy and empathy from the app, *as if it cares*. One participant would like to have this app available on a mobile device instead of a computer.

### Attitudes Toward Using Technology in Health Management After Actual Use

Participants were given technology to monitor their weight (parameter from nutritional domain), physical activity (physical function domain), and daily positive emotions (well-being domain) for a period of 4 weeks. The individual interviews performed after this period revealed that all participants were satisfied with using technology to monitor at least one health domain.

Weight monitoring was the favorite feature for 3 out of 11 participants. These participants said that the fact that the app stored the weight measurements automatically and provided an overview over time was very positive, as it saved time when compared with the conventional procedure of registering the weight with pen and paper. These 3 participants were those who showed a more positive attitude regarding monitoring of nutrition on a daily basis, as they had already been doing it upon request from their therapists.

Despite the moderate interest shown regarding the use of technology to monitor physical activity in daily life during the first interviews (only 6 participants said they would like to use an app in daily life), after the 4 weeks of the study, all participants reported a positive experience. Overall, 9 participants mentioned experiencing an added value with this feature and would like to keep using it, whereas the 2 other participants said that, although the idea was interesting, they would not use the monitoring system in daily life as they know they are more active than the general population of the same age. In addition, 8 participants mentioned that they became more active during the 4-week period, and 5 interviewees mentioned that they became more aware of their daily physical activity:

I find it a piece of art, in fact, that this is possible [...] Because it does make you aware of things that you don’t really think about.

67-year-old female

The attitudes toward monitoring well-being on a daily basis changed less than in the physical function domain. After using the technology for 4 weeks, only 4 participants perceived an added value for monitoring well-being in their daily life. Nevertheless, 6 participants reported becoming more aware of their own well-being after using the technology. The most important reason was that they were invited to reflect on questions that they would not do by themselves. However, as the questions were the same every day, after a short period, the reflective effect vanished and most participants reported answering the question almost automatically. Furthermore, the low interest in monitoring well-being after using the technology for 4 weeks was influenced by the fact that, contrarily to what happened with weight and physical activity, older adults were not provided any feedback or overview on their answers.

## Discussion

### Principal Findings

This study explored current practices in health management, attitudes toward monitoring health in daily life supported by technology, and wishes of technology from the perspective of community-dwelling older adults. Moreover, we investigated whether the attitudes toward technology supporting health management changed after actual use of monitoring technology in daily life. The older adults in our study were in general engaged in their health management, particularly on the physical domain. Furthermore, the older adults were willing to use technology in daily life to monitor their health and to help them in the adoption of healthier behaviors, as long as they perceived the technology was tailored to their needs. However, the wishes of technology differed per health domain. In the nutritional and physical domains, older adults search for technology that creates awareness about current behaviors and coaches them in the adoption of healthier behaviors. Contrarily, for the cognitive function, older adults look for a training system but do not want to receive feedback on current status or an overview of changes over time. Furthermore, when developing technology to be used in daily life, not only the wishes should be considered but also the fears that the older adults state concerning technology, such as the replacement of human contact. In the next paragraphs, based on the results of our study, we provide a set of recommendations for those interested in the development and implementation of technology-based interventions to prevent functional decline in the daily lives of older adults.

### Current Practices in Health Management

Although actively engaged in their health management, the older adults participating in our study were not always confident, or even right, about what they believed as being healthy or not. Moreover, older adults wish to obtain meaningful information about how and why they should change a current behavior. For example, when openly asked about general practices in health management, older adults primarily thought about the physical domain. Contrarily, none of the interviewees mentioned cognitive function, as also reported in the study by Menichetti and Graffigna [26]. This means that older adults are themselves not aware of the holistic dimension of functioning. Moreover, in our study, cognitive function was the most difficult health domain to talk about. Older adults did not understand what cognitive function was or their knowledge was limited to memory-related issues. Despite the fact that older adults have shown a better understanding about the physical domain, there are still misconceptions. For instance, it is not clear that being active goes beyond the practice of structured exercise. Interventions should make individuals aware that all daily movements count by promoting an active lifestyle beyond the motivation for physical exercise, as in the studies by Tabak et al and Fanning et al [22,27]. In the nutritional domain, beliefs on what is (un)healthy are affected by cultural traditions (eg, bread as the core element of all meals in a day in the Netherlands). *All-in-all, improvement of health literacy must be prioritized when aiming to prevent functional decline.*

In our study, older adults tend to adopt a functional perspective of health. This is in line with previous research suggesting that, as people grow old, the conceptualization of being healthy changes from *disease avoidance* to *being able to do daily activities independently* [1,28]. Further research should investigate the effectiveness of interventions in which technology supports the individual in reaching personalized goals related to daily activities. As an example, one can think about the goal: “I want to be able to pick up my grandchildren from school.” Technology could then support in maintaining or achieving the skills needed from the different health domains to keep doing this activity independently. Therefore, *technology to support prevention of functional decline must go beyond the disease-oriented perspective and focus, instead, on strategies to maintain independence on daily activities.*

### Attitudes Toward Using Technology in Health Management

Older adults were in general positive toward using technology in health management on a daily basis. However, technology should provide this support without interfering with the daily activities and without consuming too much time. This can be achieved with unobtrusive sensing and easy communication between individual and technology. Another general concern mentioned was the fear that, through the use of technology, older adults would listen less to the signals of their own bodies. In fact, technology can support and increase functioning, but it can also diminish capabilities through disuse [6,29]. Technology must then keep challenging older adults to use and improve their abilities instead of being a simple facilitator. Finally, the interviewees shared the fear that technology would replace human contact, as also reported by Peek et al [13]. In this case, technology can support communication between older adults in both real and virtual worlds. *Fears related to technology that deserve attention are (1) technology as attention thief in daily life, (2) technology leading to diminish abilities through disuse, and (3) technology as replacement for human contact.*

Older adults wish to perceive the technology as tailored to their own wishes, disabilities, and preferences. The World Health Organization identified *diversity in older age* as a challenge when developing policies targeting the promotion of healthy aging [1]. The same challenges serve for technology. Designers and technology developers should take that into consideration and design modular apps that allow older adults to enable or disable functionalities according to their personal needs and wishes. In this way, older adults also perceive as being in control over the technology, instead of feeling that they are being controlled by technology. For example, older adults must be given the possibility to decide whether they want to share their information with other people or not. Interventions to support prevention of functional decline should also be tailored in terms of the motivational messages generated (eg, based on current stage of change from the transtheoretical model [30]) or in terms of the strategy how the training is provided to the individuals (eg, gamified training of cognitive function vs reading challenging texts, based on individual’s preference). Another possibility is suggested by Menichetti and Graffigna who define 3 experiential positions regarding health management: locked position, awakening position, and climbing position [26]. Technology might play different roles in each one of the experiential positions. In the first, it can go more in the health literacy direction; in the awakening position, technology might help setting up plans as daily tasks; and finally in the climbing position, technology can support the maintenance of good practices. In conclusion, *a one-size-fits-all approach is not possible in the use of technology to support prevention of functional decline.*

To talk about their health and, in particular, about cognitive function and well-being, older adults wish to feel that *the technology cares for them*. In fact, exploring wishes of technology to monitor well-being in daily life became extremely difficult as most of the older adults participating in this study did not see well-being as a component of their health or as something that can be monitored and trained over time. *Technology should show empathy and sympathy.*

Older adults would like to be given the opportunity to share their knowledge and experience with peers. Other social interactions of interest would be sharing experiences and nudging and congratulating each other. The need of a moderator could be avoided by creating closed groups where information is only accessible by friends. In this way, older adults would like to have an active role not only in the development of the app but also during its use. *Technology should provide the opportunity for older adults to share their own knowledge and experiences with peers.*

After using technology to monitor physical activity for a period of 1 month, all participants recognized the value of it, supporting the hypothesis that attitudes toward technology might change after a short period of use, as suggested previously [11-13]. Weight tracking in daily life is a procedure that people are already familiar with, as most people already do it on a regular basis. The advantage of technology is that it stores the results automatically and provides an overview over time. Well-being was from the beginning the health domain that participants were more skeptical about. This attitude barely changed after the study period. We strongly encourage researchers to perform similar studies but start with health literacy aimed to break the prejudice against well-being, as there is growing evidence that higher experience of positive emotions is not only associated with better physiological markers of health [31-33] but also with better functioning [34].

### Strengths and Limitations

This study extends the work of Yusif et al, de Veer et al, and Peek et al [4,13] by taking a holistic perspective of health, without a design of a specific product in mind. The approach taken was “Imagine everything is possible, what would you like to see.” This is strength of our study compared with existing literature as it does not limit the mindset of the participants.

The aim of this qualitative study was to develop a deep understanding of the health management practices in daily life and how these relate to wishes with respect to technology among older adults. To reach this aim, in-depth individual interviews (taking approximately 1 hour 30 min to 2 hours per participant) were conducted to get a deep understanding of the principles and values of each participant. In line with the prevailing view on qualitative research sample sizes in seminal papers on this point, we believe that the sample size is in line with the objectives of this study [1-3]. Through in-depth conversations with 12 older adults, we obtained a richness and depth of explorations and descriptions that would likely not be possible with shorter interviews with a larger population.

The interviews were part of a larger study regarding monitoring of health with mobile technology. The participants in this study were aware that they would receive ambulatory technology to monitor physical activity, weight, and daily well-being on a daily basis. Therefore, they were intrinsically motivated to use technology; otherwise, they would have not participated in the study. In this way, the data collected regarding attitudes toward monitoring physical activity might be biased as the participants were a priori interested in monitoring their physical activity; otherwise, they would not participate in the study. However, as we are thinking about technology for a population with no or mild limitations, we are exactly targeting people that have some sort of intrinsic motivations, instead of being told to use technology by a health care professional.

Most of the interviewees were not aware of the possibilities provided by technology and in general needed several hints to come up with suggestions, also reported in other studies [13,35]. The hints provided might have biased the results on the wishes from technology; however, not giving hints would make it impossible to have a conversation on the topic.

### Conclusions

In this study, we explored (1) current practices in health management, (2) attitudes toward using technology in health management, (3) wishes of technology, and (4) changes in attitudes toward technology after actual use in daily life. On the basis of interviews with community-dwelling older adults before and after using technology, we conclude that older adults do wish to use technology in daily life to support them in managing their health in the prevention of functional decline, particularly in the nutritional, cognitive, and physical domains. Contrarily, well-being was not perceived as a health domain or it was not clear how technology can be of any support. Attitudes toward using technology in daily life only changed in the physical domain, but noticeably, with all participants perceiving an added value after use. We summarize the results of our study in a set of recommendations to researchers, clinicians, and all those interested in developing and implementing technology-based interventions in the daily life of older adults to support prevention of functional decline. Further research should investigate whether the proposed strategies improve adherence to interventions deployed in the daily life of older adults.

## Abbreviations

PERSSILAA: Personalised ICT Supported Service for Independent Living and Healthy Ageing
